# Association of Meconium-Stained Amniotic Fluid and Histological Chorioamnionitis with Fetal Inflammatory Response in Preterm Deliveries

**DOI:** 10.3390/children12040477

**Published:** 2025-04-07

**Authors:** Dóra Csenge Balogh, Kinga Kovács, Őzike Zsuzsanna Kovács, Eszter Regős, Attila Fintha, Ágnes Harmath, Miklós Szabó, Ákos Gasparics, Péter Varga

**Affiliations:** 1 Department of Obstetrics and Gynecology, Semmelweis University, H-1088 Budapest, Hungary; balogh.dora1@semmelweis.hu (D.C.B.); kingakovacs3@gmail.com (K.K.); kozike7@gmail.com (Ő.Z.K.); harmath.agnes@semmelweis.hu (Á.H.); gasparics.akos@semmelweis.hu (Á.G.); 2 Department of Pathology and Experimental Cancer Research, Semmelweis University, H-1088 Budapest, Hungary; regos.eszter@semmelweis.hu (E.R.); fintha.attila@semmelweis.hu (A.F.); 3 Department of Neonatology, Pediatric Center Semmelweis University, 53-54 Bokay Janos St 1083, H-1088 Budapest, Hungary; szabo.miklos@semmelweis.hu

**Keywords:** meconium, preterm, MSAF, chorioamnionitis, FIR, intrauterine inflammation

## Abstract

Background: The importance and etiology of meconium-stained amniotic fluid (MSAF) in preterm pregnancies are still poorly understood. Among other factors, intrauterine inflammation is proposed to be a pathophysiological change associated with MSAF. To study the extent of intrauterine inflammation, histological evaluation represents the “gold standard” of diagnostics. Objectives: To investigate the concomitant occurrence of MSAF and histological chorioamnionitis (HCA) and fetal inflammatory response (FIR). To investigate the incidence of short-term neonatal outcomes in preterm infants born from MSAF. Materials and methods: We conducted a single-center retrospective study in a tertiary neonatal intensive care unit between 2020 and 2022. 237 preterm infants born ≤ 32 weeks or with ≤1500 g birthweight were investigated. The group of infants born from MSAF was compared to the group of infants born from clear amniotic fluid (CAF). The variables measured were the following: HCA, FIR, maternal and fetal vascular malformations (MVM, FVM), maternal clinical and laboratory signs of chorioamnionitis (CA), early neonatal outcomes, neonatal white blood cell count (WBC) in the first day of life, and neonatal c-reactive protein (CRP) level on the second day of life. Histological evaluation of the placenta and the umbilical cord was based on the recommendation of the 2014 Amsterdam Placental Workshop Group Consensus Statement (APWGCS). Results: Out of 237 preterm infants (mean gestational age: 28.6 (95% CI: 28.2; 28.9) weeks, mean birth weight: 1165 (95% CI: 1110; 1218) grams), 22 were born from MSAF. There was no difference between the perinatal characteristics of the two groups. A higher incidence of HCA (54.5% vs. 32.6%; *p*: <0.001), a higher incidence of stage 3 HCA (45.4% vs. 9.3%), a higher incidence of FIR (50% vs. 16.7%; *p*: <0.001), and a higher incidence of stage 3 FIR (18.2% vs. 1.9%) were found in the MSAF group in comparison with the CAF group. A higher incidence of elevated (>30 mg/L) maternal CRP level (36.8% vs. 15.3%; *p*: 0.02) and elevated (>15 mg/L) neonatal CRP level (31.8% vs. 14.4%; *p*: 0.03) was detected in the MSAF group. Among neonatal complications, severe (Stage III/IV) intraventricular hemorrhage (IVH) had a higher incidence in the MSAF group (22.2% vs. 5.1%; *p*: 0.005). Conclusion: MSAF in preterm pregnancies is associated with a severe maternal and fetal inflammatory response in the placenta and the umbilical cord. MSAF is also accompanied by elevated systemic inflammatory parameters and a higher incidence of severe neonatal IVH as well.

## 1. Introduction

Meconium-stained amniotic fluid (MSAF) occurring in term and post-term neonates is a well-described entity with an incidence estimated between 9.2 and 17% [1,2]. In term deliveries, MSAF is proposed to be the result of acute intrauterine/peripartum hypoxia [3]. It has been stated in numerous studies that meconium and meconium-related conditions (e.g., meconium aspiration syndrome, asphyxia) are strongly associated with adverse neonatal outcomes, including both short-term consequences and long-term neurological sequelae [1,4].

### 1.1. MSAF and Preterm Pregnancies

In contrast, the etiology and interpretation of MSAF found in preterm pregnancies are still poorly understood, and the majority of the studies dealing with this phenomenon present low case numbers and/or were published decades ago [5]. The incidence of meconium-stained amniotic fluid increases with advancing gestational age, connected to the maturation of the fetal gastrointestinal system. In the gastrointestinal system of the fetus, meconium appears at around 12 weeks of gestation; the volume of meconium increases during the third trimester. However, immature fetuses rarely pass meconium into the amniotic sac due to decreased intestinal peristalsis, increased muscle tone of the anal sphincter, and low motilin levels [6,7]. Even in the preterm population, a difference can be detected in the frequency of MSAF: whilst the frequency of MSAF is above 5% in the population of neonates born at 36 weeks, it is between 4 and 4.7% in neonates born at 32–35 weeks and 3.7% in neonates born at 31 weeks [5,8,9,10].

Unlike term neonates, in the preterm population, MSAF is not considered unequivocally as a marker of hypoxia [5]. It has been observed that MSAF in preterm deliveries is associated with premature rupture of membranes and adverse neonatal outcomes as well, e.g., necrotizing enterocolitis (NEC) [11], severe intraventricular hemorrhage (IVH) [10], and cerebral palsy (CP) [12].

### 1.2. MSAF and Intrauterine Inflammation

As a trigger, several pathophysiological mechanisms were proposed as the origin of meconium in the amniotic fluid: intrauterine infection, bacterial contamination, Listeria infection, cord compression, and the presence of maternal and/or fetal inflammatory response; intrauterine inflammation [13,14,15,16]. Previous studies have found an association between MSAF in preterm births and histologically confirmed chorioamnionitis (CA), although these studies did not use internationally accepted uniform histological criteria for the diagnosis of CA [17,18].

### 1.3. Aim of Our Study

The focus of the present study was to investigate the association between intrauterine inflammation and MSAF among preterm infants. In the diagnostics of chorioamnionitis, histological evaluation represents the gold standard. Our knowledge, our study is the first research on the significance of meconium-stained amniotic fluid among preterm infants in which the diagnosis of histological chorioamnionitis was based on a standardized, international guideline. We evaluated the inflammatory state of the placenta and the umbilical cord of preterm infants born from MSAF—based on the standardized recommendation of the Amsterdam Placental Workshop Group Consensus Statement (APWGCS) [19,20].

In addition, we studied the maternal and neonatal inflammatory markers and the incidence of early neonatal morbidities.

## 2. Methods

**Figure 1:**
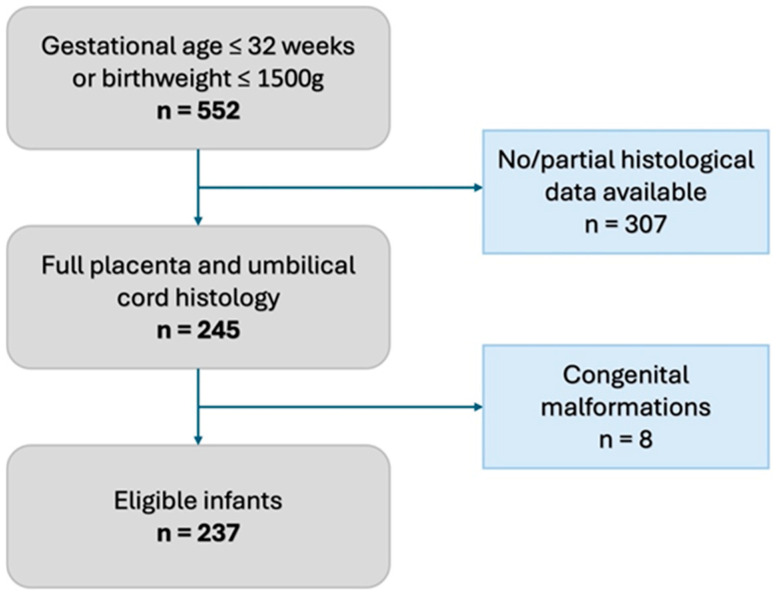
Flowchart of inclusion criteria.

We conducted a single-center retrospective observational study in a tertiary Neonatal Intensive Care Unit of the Baross Street Department of Obstetrics and Gynecology, Semmelweis University, Budapest, between January 2020 and December 2022. According to our institutional protocol, every preterm infant born ≤ 32 gestational weeks or with a birthweight ≤ 1500 g receives a complete placenta and umbilical cord histopathological evaluation based on the APWGCS guideline. We excluded neonates who did not have a complete histopathological evaluation and those who were born with congenital malformations (Figure 1).

We divided the study subjects into two groups based on the presence of meconium in their amniotic fluid. The presence or absence of meconium was judged by the attending obstetrician.

We compared the results of the histological examinations, the perinatal characteristics, the complications of surviving infants, and maternal and neonatal laboratory parameters of the meconium-stained amniotic fluid (MSAF) and the clear amniotic fluid (CAF) group.

### 2.1. Histopathology

Histological evaluation of the placenta and the umbilical cord took place at the 1st Department of Pathology, Semmelweis University, based on the recommendation of the APWGCS. We assessed the incidence of histological chorioamnionitis (HCA), fetal inflammatory response (FIR), and maternal and fetal vascular malformations (MVM, FVM). The same two pathologists (E.R. and A.F.) conducted the histological evaluation.

### 2.2. Perinatal Data

We collected and evaluated the following perinatal parameters: gestational age at birth, birth weight, small for gestational age (SGA), gender, singleton/multiple gestation pregnancy, cesarean section/vaginal delivery, premature rupture of membranes, antenatal steroid course, and Apgar scores at 1 min and 5 min.

SGA (small for gestational age) was defined as birth weight lower than the 10th percentile for gestational age (Fenton preterm growth chart) [21]. A complete course of antenatal corticosteroid prophylaxis was defined as steroid treatment started at least 48 h before the delivery; a partial course of antenatal prophylaxis was defined as steroid treatment started at least 2 h before the delivery. In the absence of steroid treatment or if the treatment was started less than 2 h before delivery, it was classified as no steroid treatment.

### 2.3. Outcomes

We studied the following complications among surviving infants: need for surfactant administration, need for mechanical ventilation, need for patent ductus arteriosus (PDA) closure (pharmacological or surgical), NEC, and severe (Bell stage III) NEC. Complications affecting the central nervous system were the following: IVH, severe (Papile III-IV stage) IVH, and periventricular leukomalacia (PVL).

We assessed maternal C-reactive protein (CRP) level and white blood cell count (WBC) before the delivery (within 72 h prior to delivery). We assessed the neonatal WBC on the first day of life and the neonatal CRP level within 24–48 h of life. We compared the mean neonatal and maternal WBC in the two groups. The cut-off level of CRP was given as ≤15 mg/L according to local protocol. We measured the incidence of elevated maternal and neonatal (>15 mg/L) CRP levels in the two groups and observed the incidence of highly elevated (>30 mg/L) maternal CRP levels as well. The CRP level was measured by our laboratory Siemens Atellica Solution laboratory automation system, and the blood count was measured with a Sysmex blood count automation system.

Survival was defined as the hospital discharge of a clinically stable infant to a level II unit. Most of our patients were transferred before the 36th postconceptional week; thus, our data are not sufficient to properly identify complications such as bronchopulmonary dysplasia (BPD), retinopathy of prematurity (ROP), or CP.

### 2.4. Statistical Analysis

In the case of variables measured in an ordinal scale, the median and interquartile range (IQR) were shown, and the difference between the groups was described by the Mann–Whitney U test. The categorical variables were described by frequency along with their 95% confidence interval (95%CI). For categorical variables, two-sided 95% Wald confidence intervals were estimated; the difference between the groups was described by a chi-square test. For continuous endpoints, the mean and the 95% confidence interval were displayed. For parameters with continuous endpoints, we applied the F test. If the variances in the two groups were equal, the difference between the groups was described by the independent samples *t*-test. If the variances in the two groups were not equal, the difference between the groups was described by the Welch test. A difference was considered statistically significant if the *p*-value was below 0.05 and the 95% confidence intervals were not overlapping. When the 95% confidence intervals were slightly overlapping, but the *p*-value was below 0.05 (due to the modest sample size), the difference was considered to be tendentious.

## 3. Results

Within the 2020–2022 period, 245 infants had full histopathological placental and umbilical cord evaluation. After the exclusion of 8 infants with congenital malformations (Figure 1, Supplementary Table S1), 237 infants were enrolled in the study, of whom 22 had meconium-stained amniotic fluid. The incidence of meconium-stained amniotic fluid was 9.3 (95% CI: 6.2; 13.7) % in the study population.

### 3.1. Perinatal Characteristics

Analyzing the perinatal characteristics of the MSAF and CAF groups, the average gestational age at delivery (27.6 (95% CI: 26.4; 28.9) weeks vs. 28.7 (95% CI: 28.3; 29) weeks (*p*: 0.11)), mean birth weight (1074 (95% CI: 889; 1258) grams vs. 1174 (95% CI: 1116; 1231) grams (*p*: 0.29)), and the survival rate (86.4 (95% CI: 66.7; 95.3) % vs. 89.3 (95% CI: 84.5; 92.8) % (*p*: 0.67)) were not different in the two groups. In addition, 9 out of 237 infants are in the moderately preterm population; all of these infants were in the CAF group. Male-to-female ratio, rate of multiple births (twins), antenatal steroid administration, frequency of cesarean section, frequency of premature rupture of membranes, and Apgar scores assessed in the 1st and 5th minute were not statistically different between the MSAF and CAF groups (Table 1).

### 3.2. Histological Findings

**Figure 2:**
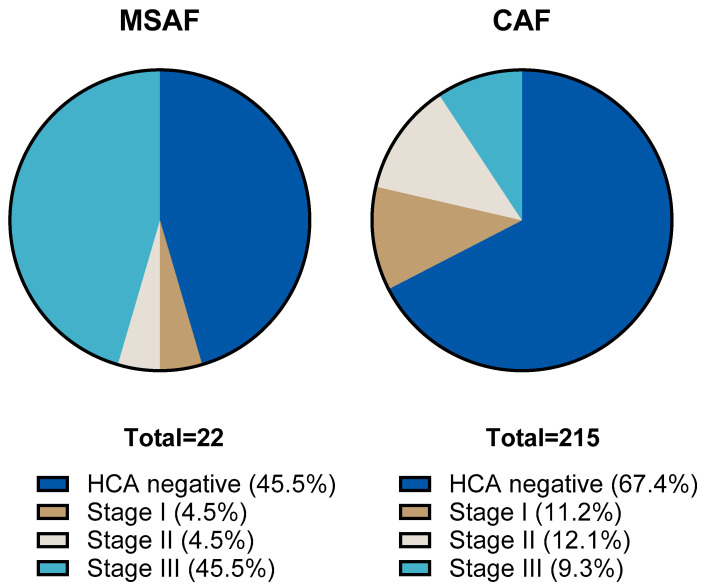
The proportion of histological chorioamnionitis (HCA) stages in the meconium-stained amniotic fluid (MSAF) and clear amniotic fluid (CAF) groups.

**Figure 3:**
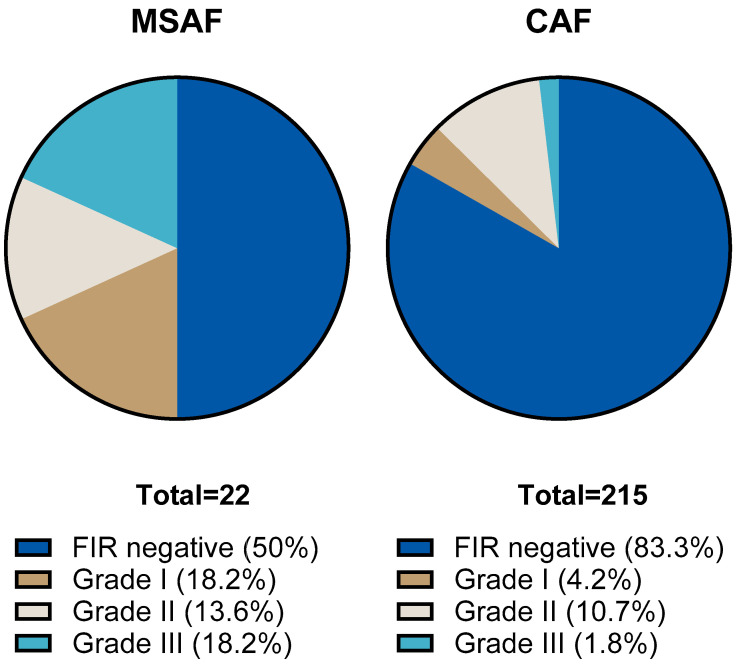
The proportion of fetal inflammatory response (FIR) stages in the meconium-stained amniotic fluid (MSAF) and clear amniotic fluid (CAF) groups.

We compared the results of placental histological examinations in the two groups. The rate of histological chorioamnionitis (HCA) grade I-III was significantly higher: 54.5 (95% CI: 34.7; 73.1) % in the MSAF group vs. 32.6 (95% CI: 26.6; 39.1) % in the CAF group (*p*: < 0.001); the greatest difference was in the rate of grade III HCA, 45.4 (95% CI: 26.7; 65.4) % in the MSAF group vs. 9.3 (95% CI: 6.7; 15.4) % in the CAF group. We also found a significantly higher frequency of fetal inflammatory response (FIR) grade I-III, 50 (95% CI: 30.1; 69.3) % in the MSAF group vs. 16.7 (95% CI: 12.3; 22.3) % in the CAF group (*p*: < 0.001); the greatest difference was in the rate of grade III FIR, 18.2 (95% CI: 6.7; 39.1) % in the MSAF group vs. 1.9 (95% CI: 0.6; 4.7) % in the CAF group (Figure 2 and Figure 3). The rates of maternal and fetal vascular malformations were not different in the two groups (Table 2).

### 3.3. Laboratory Parameters

We investigated the incidence of elevated neonatal and maternal CRP levels and the mean maternal and neonatal WBC. The rate of elevated (>15 mg/L) neonatal CRP level, 31.8 (95% CI: 16.2; 52.9) % vs. 14.4 (95% CI: 10.3; 19.9) % *p*: 0.03, and the rate of highly elevated (>30 mg/L) maternal CRP, level 36.8 (95% CI: 19.1; 59.1) % vs. 15.3 (95% CI: 10.4; 21.8) % *p*: 0.02, were tendentially higher in the MSAF group. Neither the rate of elevated maternal (>15 mg/L) CRP level nor the mean maternal and neonatal WBC levels were different in the two groups (Table 3).

### 3.4. Neonatal Outcomes

The incidence of IVH and PVL was not statistically different among survivors in the two groups. The incidence of severe IVH was higher, 22.2 (95% CI: 8.5; 45.8) % vs. 5.1 (95% CI: 2.6; 9.6) % (*p*: 0.006), in the surviving infants of the MSAF group. There was no significant difference in the need for mechanical ventilation, surfactant therapy, and pharmacological or surgical PDA closure. The incidence of NEC and severe NEC was not different in the surviving infants of the two groups (Table 4).

## 4. Discussion

### 4.1. MSAF and the Diagnosis of Chorioamnionitis

It has been presumed that meconium in the early-preterm population is associated with intrauterine infection and clinical chorioamnionitis [16,22]. It has been reported that the presence of MSAF is an independent risk factor of peripartum maternal bacteremia [23] and has also been found to be associated with premature rupture of membranes [10]. In preterm deliveries, the presence of meconium is correlated with a significantly higher rate of chorioamnionitis and positive cultures [18].

The clinical signs of chorioamnionitis were described by Gibbs 30 years ago [24]. Based on clinical symptoms only, 50% of CA cases can be proven. Several attempts were made to reach for a more precise prenatal diagnosis [25,26,27]; however, a “gold standard” of prenatal diagnostics is still missing. The final diagnosis of chorioamnionitis can be made by histopathological evaluation [28].

The focus and strength of our study was the strict histopathological evaluation of the placenta and the umbilical cord. In the past, these evaluations were based on local protocols [17,18]. Using the 2014 APWGCS criteria, we found a correlation between severe intrauterine inflammation and MSAF, consistent with their studies.

### 4.2. Histological Evaluation

The important finding of our study, a higher rate of HCA in the MSAF group, is in accordance with the literature data [18,29]. In addition, as a new observation, we found a higher rate of FIR in the MSAF group as well. Examining the placenta of term and near-term preterm infants, HCA or FIR was observed more often in those born from MSAF [17]. In these studies, the histological examination of the placenta was not performed according to uniform internationally defined criteria. The diagnosis of HCA and FIR was based on the individual judgment of the pathologist, and the severity of the inflammation was not classified [17,18,29]. In our study the histological examination was carried out uniformly according to the recommendation of the 2014 APWGCS. The APWGCS recommendation distinguishes three stages of HCA and FIR based on the severity of the inflammation; thus, we were able to examine not only the presence of chorioamnionitis and FIR but also its severity.

In the MSAF group, the ratio of the most severe stage of HCA and the most severe stage of FIR was significantly increased compared to the group with CAF. As a novel observation, it is important that MSAF is associated with an advanced inflammatory response in preterm infants, as the severity of intrauterine inflammation might worsen neonatal outcomes as well [30].

### 4.3. Laboratory Findings

Intrauterine inflammation is associated with leukocytosis and elevated maternal and neonatal CRP values. Sensitivity and specificity for maternal CRP (≥20 mg/L) were 59% and 83% [31], respectively; therefore, it is not an internationally accepted marker of CCA. On the contrary, neonatal CRP is widely used in the diagnosis of neonatal sepsis. CRP levels begin to increase after 10–12 h of the onset of inflammation and peak at around 48 h. Due to the delayed increase in CRP levels as a response to infection, CRP has low sensitivity within the first day of life [32]. Along with a higher rate of histological inflammation, we observed a tendentially higher frequency of elevated neonatal CRP levels (on the 2nd day of life) and highly elevated maternal CRP levels as well.

Leukocytosis with maternal fever confirms clinical chorioamnionitis [33]. The normal value of the neonatal WBC in the first hours of life is difficult to determine, and elevated WBC does not correlate with EOS [34]. We did not find any difference either in the maternal or in the neonatal mean WBC between the MSAF and CAF groups.

### 4.4. Neonatal Outcomes

Higher rates of intrapartum and postpartum mortality [22] and higher rates of IVH [10] and NEC [11] were found in preterm infants born from MSAF. Similarly, we found a higher incidence of severe IVH in the MSAF group, despite the moderate number of included preterm neonates and low frequency of MSAF cases. On the contrary, we found no difference regarding the other investigated outcomes.

Intrauterine inflammation is associated with a poor outcome, a higher rate of neonatal sepsis, IVH, PVL, BPD, and NEC [35]. There is evidence of the disturbing effect of intrauterine inflammation on brain development [36,37] in preterm pregnancies. The role of meconium and its proinflammatory properties in term infants and MAS was studied extensively [38], but not in preterm MSAF pregnancies. The higher rate of severe IVH in the MSAF group might be caused by severe fetal inflammation, but MSAF could be an independent risk factor as well. Due to the low case number and the retrospective design of our study, further targeted studies are necessary to distinguish between them.

### 4.5. Clinical Significance

The most common cause of preterm premature rupture of membranes (PPROM) is intrauterine inflammation. In our study we found that almost half of the preterm infants in the MSAF group had the most severe form of HCA, and also in half of the cases HCA was accompanied by FIR. Based on these findings, MSAF could be a simple and easily detectable sign of advanced intrauterine inflammation. The obstetric management of PPROM is still debated according to the literature [39,40,41], and MSAF might also be a factor in decision-making.

### 4.6. Limitations of the Present Study, and Future Directions

As we conducted a retrospective analysis, not all the clinical data were available. To gain a better understanding of chorioamnionitis and MSAF, a prospective study would be necessary with accurately documented clinical symptoms and unified laboratory and microbiological sampling.

Similarly to other studies dealing with MSAF in preterm populations, the presence of meconium was based on clinical judgment [10,18,22]. The clinical judgment of MSAF might be misleading in some cases, and histologic confirmation of meconium during the examination of the placenta is also complicated—especially if the amniotic fluid has been meconium-stained for a short time [42]. A few solutions for precise meconium detection were tried recently: “meconium-crit” determination, spectrophotometry [9], or immunohistochemical detection of ZnCP-I during histological evaluation [43].

Gluck et al. noted the meconium thickness level correlates with the adversity of the neonatal outcome. In our database, the level of meconium thickness is not recorded [1].

A limitation of our study could be the heterogeneity regarding gestational age in our preterm population. As our research included infants born ≤ 32 gestational weeks and/or with a birthweight ≤ 1500 g, a few of our patients are in the moderately preterm population. However, in our population the number of these individuals reaches 9 (out of 237), making it a relatively small percentage of our preterm population.

Out of the 237 preterm infants, only 22 infants were born with MSAF. The low case number in the MSAF group makes it harder to draw a conclusion. MSAF is present in a small proportion of preterm deliveries; therefore, higher case numbers are required to properly investigate the consequences of MSAF.

## 5. Conclusions

Higher rates of intrauterine inflammation and severe intrauterine inflammation occurred more often in the MSAF group. The incidence of severe IVH was also higher in this group of preterm infants. Based on our study, MSAF appears to be an unfavorable prognostic factor for preterm infants. Regarding the low case number of infants in the MSAF, further research is needed to clarify the significance of MSAF.

## Figures and Tables

**Table 1:** Perinatal factors in the meconium-stained amniotic fluid (MSAF) and the clear amniotic fluid (CAF) group.

	MSAF Group n = 22	CAF Group n = 215	*p*
Survival, % (95% CI)	86.4 (66.7; 95.3)	89.3 (84.5; 92.8)	0.67
Gestational age (weeks), mean (95% CI)	27.6 (26.4; 28.9)	28.7 (28.3; 29)	0.11
Birth weight (g), mean (95% CI)	1074 (889; 1258)	1174 (1116; 1231)	0.29
SGA % (95% CI)	18.2 (6.7; 39.1)	13.9 (9.9; 19.2)	0.59
Partial course of antenatal corticosteroid therapy, % (95% CI)	91 (72.2; 97.5)	92.5 (88.2; 95.4)	0.78
Complete course of antenatal corticosteroid therapy, % (95% CI) any	54.6 (34.7; 73.1)	56.1 (49.4; 62.6)	0.89
PPROM, % (95% CI)	22.7 (10.1; 43.4)	18.1 (13.6; 23.8)	0.6
Female sex, % (95% CI)	46.5 (40; 53.2)	36.4 (19.7; 57.1)	0.36
Multiple births, % (95% CI)	13.6 (4.8; 33.3)	31.5 (25.6; 38)	0.08
Cesarean delivery, % (95% CI)	77.3 (56.6; 89.9)	88.3 (83.3; 92)	0.14
Apgar score at 1 min, median (IQR)	7 (5; 8)	9 (8; 9)	0.1
Apgar score at 5 min, median (IQR)	8 (7; 8)	9 (8; 9)	0.16

SGA: small for gestational age, PPROM: premature rupture of membranes.

**Table 2:** Histological findings of placentas in the meconium-stained amniotic fluid (MSAF) and the clear amniotic fluid (CAF) group.

	MSAF Group n = 22	CAF Group n = 215	*p*
CA grade I-III, % (95% CI)	54.5 (34.7; 73.1)	32.6 (26.6; 39.1)	<0.001
FIR grade I-III, % (95% CI)	50 (30.1; 69.3)	16.7 (12.3; 22.3)	<0.001
MVM, % (95% CI)	50 (30.1; 69.3)	48.4 (41.8; 55)	0.88
FVM, % (95% CI)	27.3 (12.8; 48.4)	13.5 (9.5; 18.7)	0.08

CA: chorioamnionitis, FIR: fetal inflammatory response, MVM: maternal vascular malperfusion, FVM: fetal vascular malperfusion.

**Table 3:** Maternal and neonatal laboratory results in the meconium-stained amniotic fluid (MSAF) and the clear amniotic fluid (CAF) group.

	MSAF Group n = 22	CAF Group n = 215	*p*
Maternal WBC (G/l) mean (95% CI)	15.5 (11.9; 19)	13.3 (12.64; 13.9)	0.22
Maternal CRP > 15 mg/L, % (95% CI)	47.4 (27.3; 68.3)	34.4 (27.4; 42.1)	0.27
Maternal CRP > 30 mg/L, % (95% CI)	36.8 (19.1; 59.1)	15.3 (10.4; 21.8)	0.02
Neonatal WBC, mean (95% CI)	11.8 (6.1; 17.5)	8.9 (8.0; 9.7)	0.28
Neonatal CRP > 15 mg/L, % (95 CI%)	31.8 (16.2; 52.9)	14.4 (10.3; 19.9)	0.03

WBC: white blood cell count, CRP: C-reactive protein.

**Table 4:** Neonatal complications of the surviving infants in the meconium-stained amniotic fluid (MSAF) group and the clear amniotic fluid (CAF) group.

	Surviving Infants in the MSAF Group n = 19	Surviving Infants in the CAF Group n = 192	*p*
Need for surfactant therapy, % (95% CI)	73.7 (50.9; 88.6)	58.3 (51.3; 65.1)	0.19
Need for mechanical ventilation, % (95% CI)	47.4 (27.3; 68.3)	29.8 (23.7; 36.7)	0.11
Need for PDA closure, % (95% CI)	26.3 (11.5; 49.1)	17.4 (12.6; 23.4)	0.12
IVH Papile I-IV st., % (95% CI)	33.3 (16.1; 56.4)	29.1 (22.9; 36.3)	0.71
Severe IVH Papile III-IV st., % (95% CI)	22.2 (8.5; 45.8)	5.1 (2.6; 9.6)	0.006
PVL, % (95% CI)	11.1 (1.9; 34.1)	6.3 (3.7; 11.8)	0.44
NEC Bell I-III st., % (95% CI)	10.5 (1.7; 32.6)	13 (8.9; 18.6)	0.76
NEC Bell III st., % (95% CI)	0 (0; 14.7)	0,5 (0; 3.2)	0.75

PDA: patent ductus arteriosus, IVH: intraventricular hemorrhage, PVL: periventricular leukomalacia, NEC: necrotizing enterocolitis.

## Data Availability

The raw data supporting the conclusions of this article will be made available by the authors on request.

## Supplementary Materials

The following supporting information can be downloaded at https://www.mdpi.com/article/10.3390/children12040477/s1, Supplementary Table S1: Excluded infants with congenital malformations.
