# Supplementary material for: Association of Meconium-Stained Amniotic Fluid and Histological Chorioamnionitis with Fetal Inflammatory Response in Preterm Deliveries
Source: Children (Basel). 2025 Apr 7;12(4):477. doi: 10.3390/children12040477 (PMC12025836; doi:10.3390/children12040477)
Supplement: Supplementary file 1 [file children-12-00477-s001.zip › children-3496973-supplementary.pdf]

Supplementary Materials:

Table S1: Excluded infants with congenital malformations

	Number of infants
Congenital diaphragmatic hernia	1
Hydrops foetalis	2
Duodenal atresia	2
Anal atresia	1
Multiplex congenital malformation	2
